# Supplementary material for: H3AGWAS: a portable workflow for genome wide association studies
Source: BMC Bioinformatics. 2022 Nov 19;23:498. doi: 10.1186/s12859-022-05034-w (PMC9675212; doi:10.1186/s12859-022-05034-w)
Supplement: Supplementary file 1 — Additional file 1. Comparison between h3agwas and BIGWAS and description and test of other scripts of workflow. [file 12859_2022_5034_MOESM1_ESM.pdf]

Supplementary matter

H3AGWAS : A portable workflow for Genome Wide Association Studies

Brandenburg *et al*

October 27, 2022

Contents

1 Overview **1**

2 Computational comparison between the BIGwas and H3AGWAS workflow **2**
- 2.1 Introduction 2
- 2.2 Data sets used 2
- 2.3 Computational setup 2
  - 2.3.1 Single machine execution 2
  - 2.3.2 Cluster execution 3
- 2.4 Comparison between association workflow of BIGwas and H3AGWAS workflow . . 4
  - 2.4.1 Data sets used and methodology 4
- 2.5 Run, troubleshooting and duration 4
- 2.6 Conclusion 4

3 Description and test of different scripts of H3AGWAS workflow : CPUs, Time **4**
- 3.1 Quality Control of genetics data 5
- 3.2 Association 9
- 3.3 Fine-mapping 9
- 3.4 Heritability 14
- 3.5 Simulations workflow 17
  - 3.5.1 Description of each workflow 17
  - 3.5.2 Simulation using 1000Genome, GWAS catalog and GCTA 17
- 3.6 Format data 18
  - 3.6.1 Convert PLINK format to VCF 18
  - 3.6.2 Convert VCF to PLINK or other format 20
  - 3.6.3 Multi-trait analyse using MTAG 24

4 Detail of experimentation in the main paper **27**

1 Overview

This supplementary note contains two substantive sections: The first give a comparison between H3AGWAS workflow and a competing tool, especially with respect to computational performance.

The second gives example runs of a number of the different workflows that we provide.

2 Computational comparison between the BIGwas and H3AGWAS workflow

2.1 Introduction

This section documents a computational comparison of the BIGwas and H3AGWAS workflow workflows in response to the findings of Kässens *et al.* that BIGwas is significantly faster than H3AGWAS workflow for QC for medium and larger files. For example, Kässens *et al.* found that data set with 5k individuals and 50k SNPs takes 8 minutes (BIGwas) versus 15 minutes (H3AGWAS workflow), and a data set with 20,554 individuals and 700k SNPs takes 135m (BIGwas) versus 537m (H3AGWAS workflow) and for even larger data sets that they could not reasonably complete execution of H3AGWAS workflow. (It must be emphasised that comparing workflows requires comparing multiple factors and in our view this is not the most important factor, but Kässens *et al* have made very serious negative findings, which we believe needs to be addressed. We do not agree with their findings)

2.2 Data sets used

The following data sets were used:

- The example data set that comes with BIGwas – this is a set based on 1000 Genomes data with 2504 individuals and 50k SNPs.
- The AWI-Gen unqc-ed data – 11062 individuals and 2.267 million SNPs.
- A simulated data set (*sim1* 22142 individuals and 2.267 million SNPs.

One difference between the two workflows is that H3AGWAS workflow expects that all the genotype data is in one PLINK file, while BIGwas allows multiple files to be input which are then merged, which exposes further parallelism. To make the comparison fair we compare the H3AGWAS workflow times with two separate runs of the BIGwas workflow:

- The BIGwas example data set is provided as two separate PLINK data sets – one the "cases" and one the "controls", each with approximately 2500 individuals. We compare the H3AGWAS workflow with the merged data setas input with the BIGwas on both the merged data and on the original split files.
- For the AWI-Gen data, we split into roughly two halves and artificially declare the two halves as cases and controls. We compare the H3AGWAS workflow on the original AWI-Gen data with BIGwas on the original data and on the split data. For the *sim1* data set a similar comparison was made.

2.3 Computational setup

We used Nextflow 21.04.1 and used Singularity with the images provided by the two tools. In both cases, both the Nextflow repos and the Singularity images were already downloaded and installed. We performed the experiments on a single machine (no scheduler) and using SLURM on a cluster.

2.3.1 Single machine execution

We used a machine with a dual Xeon Silver 4214 CPUs running at 2.20GHz (24 physical cores, 48 hyper-threaded cores) and 128GB of RAM, and all data inputs and outputs were stored on a Seagate ST2000NM0008 2TB SATA Hard Drive. The machine was otherwise unloaded.

We ran both of these on the default settings – presumably both developers chose appropriate default settings so that is fair enough. However, H3AGWAS workflow has performance parameters that the user can set if they have more powerful computers. The `max_plink_cores` parameter is

Data set	H3AGWAS workflow		BIGwas			
			Split		Merged	
	Elapsed (s)	CPU h	Elapsed (s)	CPU h	Elapsed (s)	CPU h
Example	34	0.05	480	0.1	496	0.2
AWI-Gen	1383	2.1	2576	0.8	3700	1.2
sim1	7278	12.3	29938	8.3	30240	8.4

Table 1: *Comparison between H3A*GWAS *workflow and BIGwas workflow using QC script and 3 different data sets using single machine execution*

by default set to 4 – this limits any PLINK process to use 4 cores (and makes 4 cores available and so hence counts to CPU hours whether used or not). If we run the Nextflow with the `--max_plink_cores=12`, the elapsed time for the AWI-Gen data set drops from 1383s to 750s at the cost of accounted CPU hours going to 4.2 CPU hours. This is a trade-off the user must consider. No doubt there are similar changes that could be made to the BIGwas workflow.

Note the difference in the AWI-Gen and *sim1* data set for the H3AGWAS workflow is a factor of 5.2. In principle we would expect the overall computational cost to scale quadratically with number of SNPs as the single biggest computational costs are steps which are quadratic. However as these components take longer there is less task parallelism as a proportion of the overall cost. The BIGwas workflow also scales super-quadratically but by a greater factor (our superficial observation is that the bulk of this extra cost is as at similar points in the computation).

2.3.2 Cluster execution

We tested on our production University Research Cluster: SLURM 20.11.8, CentOS7.9, Singularity 3.6.3 (default Singularity OSG release). The cluster is heterogeneous so we set Nextflow *clusterOptions* to execute only on 20 nodes with dual core Intel Xeon Silver 4114 CPUs running at 2.2GHz (20 physical, 40 hyper-threaded cores per node – note that these machines are slower than the one we tested above). Since this is a production cluster we were unable to test while the cluster otherwise completely idle but we could test late on weekend with only a few other jobs running so we do not think that this affected the results (we manually inspected that the jobs ran on machines not being used by other jobs).

Singularity issues: We were unable to run BIGwas on the cluster in its default Singularity setting. Like many production HPC systems, the cluster follows recommendations to not allow Singularity with *setuid* enabled (`https://sylabs.io/guides/latest/user-guide/security.html`). The disadvantage of this is that standard Singularity SIF images cannot be directly executed but must be copied (unsquashed) to a temporary disk for each separate Nextflow process[1]. The BIGwas image is 11GB in size in SIF (compressed) format and the computational cost of unsquashing, especially when multiple processes are doing this in parallel made running the workflow impractical. The same Singularity issue applies to H3AGWAS workflow, of course. However, the H3AGWAS workflow container design approach is to have several, specialised containers rather than one monolithic container. For example, the workhorse `py3plink` container is ≈450MB in size – so although there is a significant penalty for running H3AGWAS workflow on the cluster using Singularity compared to running the natively installed software, it is not an outrageous penalty. In order to perform the testing below, we were able to enable setuid for (this requires root privileges), but this is not something we are allowed to leave for extended periods. In many environments setuid is enabled in Singularity installations (as it was in our testing on the single machine), so many users will not face this problem. But we suspect that other Singularity users in production HPC environment will run into the same problem as us. (We also tested Singularity 3.8 and 3.9 and it had the same problem).

[1]And to be clear if a Nextflow process is executed 10 times in parallel then each instantiation of the process requires this.

Data set	H3AGWAS workflow		BIGwas			
			Split		Merged	
	Elapsed (s)	CPU h	Elapsed (s)	CPU h	Elapsed (s)	CPU h
Example	189	< 0.1	967	0.4	899	0.2
AWI-Gen	2025	3.8	3712	1.2	3647	1.1
sim1	8346	16.2	33728	8.4	31028	8.6

Table 2: *Comparison between H3A*GWAS *workflow and BIGwas workflow using the QC script and three different data sets using the Cluster and SLURM*

2.4 Comparison between association workflow of BIGwas and H3AGWAS workflow

2.4.1 Data sets used and methodology

We used output of QC produced by the BIGwas workflow to run association of H3AGWAS workflow and BIGwas. For both workflow we ran PLINK as the underlying association testing tool defaults of workflows where used. We ran each test using an Intel Xeon Silver 4114 dual core processor (40 hyper-threaded cores on 20 physical cores) with 128GB of RAM

2.5 Run, troubleshooting and duration

Table 3 shows the comparison. For the small test Data set, duration is lower for BIGwas than H3AGWAS workflow, but for bigger sample size and SNP number, H3AGWAS workflow performed better.

In using BIGwas, we observed missing data causes error on workflow when used with binary phenotype.

Data set	SNPs number	Sample Size	BIGwas	H3AGWAS workflow
Example	35317	2464	35	70
AWI-GEN	2120006	8487	2383	1637
sim1	2091083	18322	5607	2327

Table 3: Comparison between H3AGWAS workflow and BIGwas workflow using Association script and 3 different data sets

2.6 Conclusion

As we have indicated, performance is not the primary measure of a workflow because ultimately the costs depend on the underlying software used and the workflow designer can neither take too much credit nor blame for this. However, it is important to demonstrate the workflow can expose appropriate parallelism, which we believe has been demonstrated. Certainly we do not believe that the experimental evidence supports the claim that the BIGwas workflow is faster than H3AGWAS workflow.

3 Description and test of different scripts of H3AGWAS workflow : CPUs, Time

This section shows additional testing of H3AGWAS workflow on our SLURM cluster as shown. Again since the cluster is a production cluster we were only able to run it on a lightly loaded cluster not on a cluster that was idle. The purpose of this testing is to give indicative real-world costs. For the tests done, the workflow execution is shown graphically as a directed acyclic graph and

the computational cost of the individual components is shown (of course, many of the individual components can be done in parallel).

3.1 Quality Control of genetics data

- Objectives : apply a quality control on genetics data.
- Input : workflow take as input PLINK file from genomics data, phenotype, sex phenotype.
- Individual filter :
  - Apply sex control with X chromosome and sex phenotype.
  - heterozygosity control using Hardy–Weinberg equilibrium.
  - missingness
  - relatedness
- SNPs filter :
  - minor allele frequency
  - heterozygosity
  - duplicated markers
  - missingness
- Output :
  - report in pdf is produce in PDF describing each steps with different filters
  - PLINK file after quality control with frequencies distribution, hardy Weinberg equilibrium... see example 1
  - intermediate files produce by workflow.
- Test: QC workflow has been apply for 12,000 individuals with genotype using h3array positions (2.4 millions positions) using the cluster and SLURM – see the statistics in table 4.

An overview of the execution can be found in Figure 2 and the detailed computational results in Table 4.

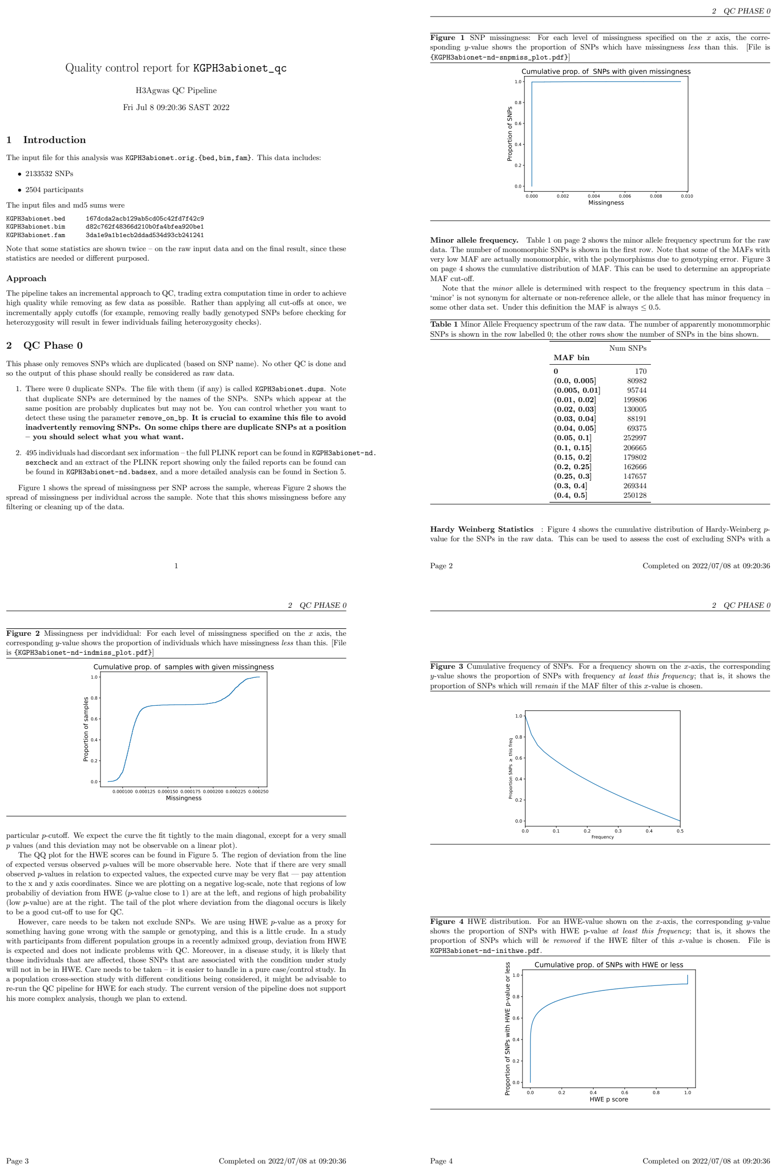

Quality control report for KGPH3abionet_qc

H3Agwas QC Pipeline

Fri Jul 8 09:20:36 SAST 2022

1 Introduction

The input file for this analysis was KGPH3abionet.orig.{bed,bim,fam}. This data includes:

- 2133532 SNPs
- 2504 participants

The input files and md5 sums were

```
KGPH3abionet.bed      167dcda2acb129ab5cd05c42fd7f42c9
KGPH3abionet.bim      d82c762f48366d210b0fa4bfea920be1
KGPH3abionet.fam      3da1e9a1b1ecb2ddad534d93cb241241
```

Note that some statistics are shown twice – on the raw input data and on the final result, since these statistics are needed or different purposed.

Approach

The pipeline takes an incremental approach to QC, trading extra computation time in order to achieve high quality while removing as few data as possible. Rather than applying all cut-offs at once, we incrementally apply cutoffs (for example, removing really badly genotyped SNPs before checking for heterozygosity will result in fewer individuals failing heterozygosity checks).

2 QC Phase 0

This phase only removes SNPs which are duplicated (based on SNP name). No other QC is done and so the output of this phase should really be considered as raw data.

1. There were 0 duplicate SNPs. The file with them (if any) is called KGPH3abionet.dups. Note that duplicate SNPs are determined by the names of the SNPs. SNPs which appear at the same position are probably duplicates but may not be. You can control whether you want to detect these using the parameter remove_on_bp. **It is crucial to examine this file to avoid inadvertently removing SNPs. On some chips there are duplicate SNPs at a position – you should select what you what want.**
2. 495 individuals had discordant sex information – the full PLINK report can be found in KGPH3abionet-nd.sexcheck and an extract of the PLINK report showing only the failed reports can be found can be found in KGPH3abionet-nd.badsex, and a more detailed analysis can be found in Section 5.

Figure 1 shows the spread of missingness per SNP across the sample, whereas Figure 2 shows the spread of missingness per individual across the sample. Note that this shows missingness before any filtering or cleaning up of the data.

1

2 QC PHASE 0

Figure 1 SNP missingness: For each level of missingness specified on the x axis, the corresponding y-value shows the proportion of SNPs which have missingness *less* than this. [File is {KGPH3abionet-nd-snpmiss_plot.pdf}]

Minor allele frequency. Table 1 on page 2 shows the minor allele frequency spectrum for the raw data. The number of monomorphic SNPs is shown in the first row. Note that some of the MAFs with very low MAF are actually monomorphic, with the polymorphisms due to genotyping error. Figure 3 on page 4 shows the cumulative distribution of MAF. This can be used to determine an appropriate MAF cut-off.

Note that the *minor* allele is determined with respect to the frequency spectrum in this data – 'minor' is not synonym for alternate or non-reference allele, or the allele that has minor frequency in some other data set. Under this definition the MAF is always ≤ 0.5.

Table 1 Minor Allele Frequency spectrum of the raw data. The number of apparently monommorphic SNPs is shown in the row labelled 0; the other rows show the number of SNPs in the bins shown.

MAF bin	Num SNPs
**0**	170
**(0.0, 0.005]**	80982
**(0.005, 0.01]**	95744
**(0.01, 0.02]**	199806
**(0.02, 0.03]**	130005
**(0.03, 0.04]**	88191
**(0.04, 0.05]**	69375
**(0.05, 0.1]**	252997
**(0.1, 0.15]**	206665
**(0.15, 0.2]**	179802
**(0.2, 0.25]**	162666
**(0.25, 0.3]**	147657
**(0.3, 0.4]**	269344
**(0.4, 0.5]**	250128

Hardy Weinberg Statistics : Figure 4 shows the cumulative distribution of Hardy-Weinberg p-value for the SNPs in the raw data. This can be used to assess the cost of excluding SNPs with a

Page 2 Completed on 2022/07/08 at 09:20:36

2 QC PHASE 0

Figure 2 Missingness per indvididual: For each level of missingness specified on the x axis, the corresponding y-value shows the proportion of individuals which have missingness *less* than this. [File is {KGPH3abionet-nd-indmiss_plot.pdf}]

particular p-cutoff. We expect the curve the fit tightly to the main diagonal, except for a very small p values (and this deviation may not be observable on a linear plot).

The QQ plot for the HWE scores can be found in Figure 5. The region of deviation from the line of expected versus observed p-values will be more observable here. Note that if there are very small observed p-values in relation to expected values, the expected curve may be very flat — pay attention to the x and y axis coordinates. Since we are plotting on a negative log-scale, note that regions of low probabiliy of deviation from HWE (p-value close to 1) are at the left, and regions of high probability (low p-value) are at the right. The tail of the plot where deviation from the diagonal occurs is likely to be a good cut-off to use for QC.

However, care needs to be taken not exclude SNPs. We are using HWE p-value as a proxy for something having gone wrong with the sample or genotyping, and this is a little crude. In a study with participants from different population groups in a recently admixed group, deviation from HWE is expected and does not indicate problems with QC. Moreover, in a disease study, it is likely that those individuals that are affected, those SNPs that are associated with the condition under study will not in be in HWE. Care needs to be taken – it is easier to handle in a pure case/control study. In a population cross-section study with different conditions being considered, it might be advisable to re-run the QC pipeline for HWE for each study. The current version of the pipeline does not support his more complex analysis, though we plan to extend.

Page 3 Completed on 2022/07/08 at 09:20:36

2 QC PHASE 0

Figure 3 Cumulative frequency of SNPs. For a frequency shown on the x-axis, the corresponding y-value shows the proportion of SNPs with frequency *at least this frequency*; that is, it shows the proportion of SNPs which will *remain* if the MAF filter of this x-value is chosen.

Figure 4 HWE distribution. For an HWE-value shown on the x-axis, the corresponding y-value shows the proportion of SNPs with HWE p-value *at least this frequency*; that is, it shows the proportion of SNPs which will *be removed* if the HWE filter of this x-value is chosen. File is KGPH3abionet-nd-inithwe.pdf.

Page 4 Completed on 2022/07/08 at 09:20:36

Figure 1: Example of four first pages of quality control report generated by pipeline

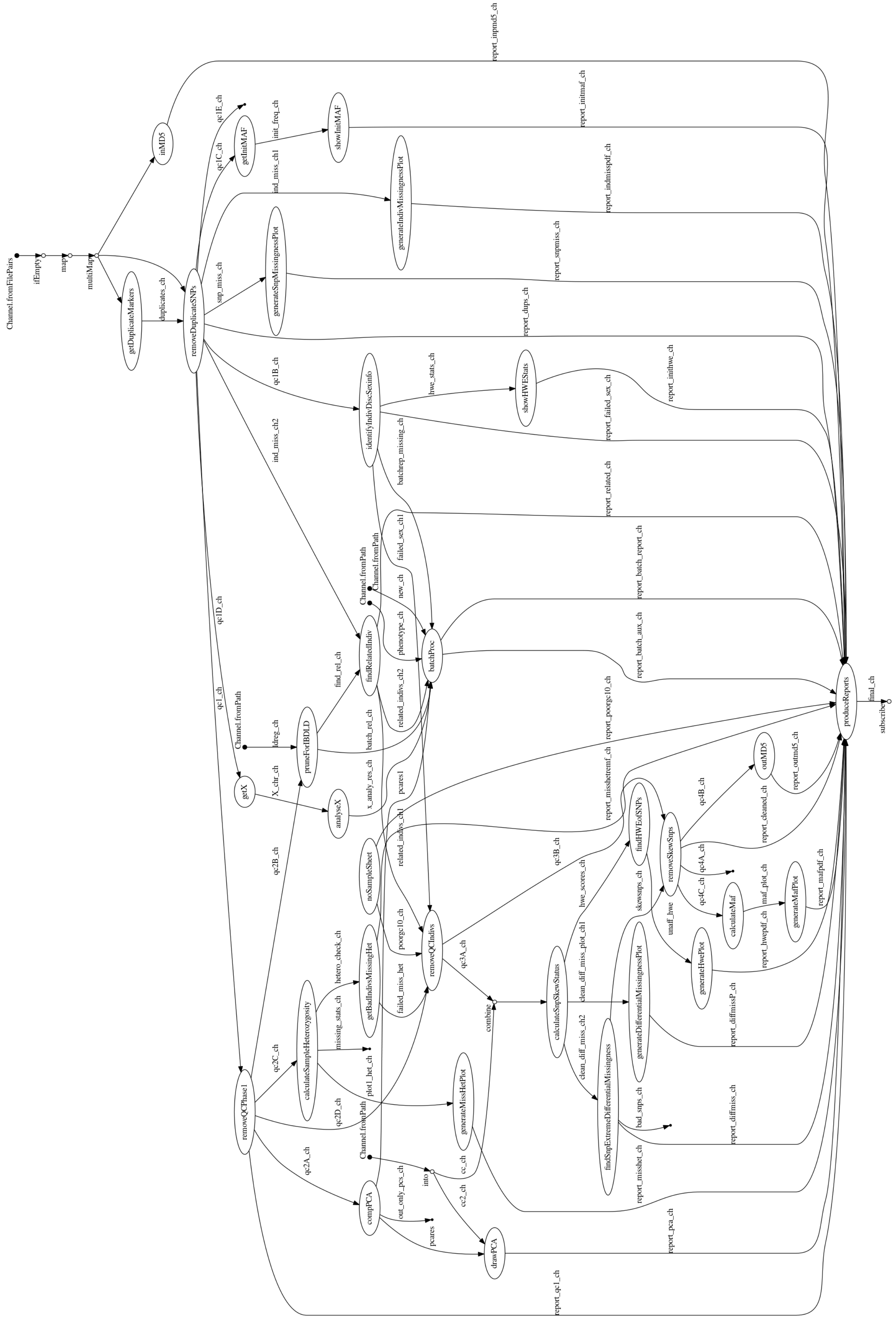
Channel.fromFilePairs
ifEmpty
map
multiMap
getDuplicateMarkers
duplicates_ch
inMD5
report_inpmd5_ch
removeDuplicateSNPs
qc1E_ch
qc1C_ch
getInitMAF
init_freq_ch
showInitMAF
report_initmaf_ch
ind_miss_ch1
generateIndivMissingnessPlot
report_indmisspdf_ch
snp_miss_ch
generateSnpMissingnessPlot
report_snpmiss_ch
report_dups_ch
qc1B_ch
identifyIndivDiscSexinfo
hwe_stats_ch
showHWEStats
report_inithwe_ch
report_failed_sex_ch
ind_miss_ch2
batchrep_missing_ch
report_related_ch
failed_sex_ch1
Channel.fromPath
Channel.fromPath
new_ch
report_batch_report_ch
qc1D_ch
phenotype_ch
findRelatedIndiv
batchProc
find_rel_ch
report_batch_aux_ch
related_indivs_ch2
qc1_ch
Channel.fromPath
idreg_ch
pruneForIBDLD
batch_rel_ch
report_poorgc10_ch
produceReports
final_ch
subscribe
getX
X_chr_ch
analyseX
x_analy_res_ch
pcares1
report_misshetremf_ch
outMD5
report_outmd5_ch
qc4B_ch
findHWEofSNPs
removeSkewSnps
report_cleaned_ch
qc3B_ch
related_indivs_ch1
noSampleSheet
qc2B_ch
qc4A_ch
qc4C_ch
calculateMaf
maf_plot_ch
generateMafPlot
report_mafpdf_ch
hwe_scores_ch
skewsnps_ch
unaff_hwe
poorgc10_ch
hetero_check_ch
getBadIndivsMissingHet
removeQCIndivs
qc3A_ch
clean_diff_miss_plot_ch1
generateDifferentialMissingnessPlot
generateHwePlot
report_hwepdf_ch
calculateSampleHeterozygosity
missing_stats_ch
failed_miss_het
combine
calculateSnpSkewStatus
report_diffmissP_ch
clean_diff_miss_ch2
plot1_het_ch
qc2C_ch
generateMissHetPlot
findSnpExtremeDifferentialMissingness
qc2D_ch
removeQCPhase1
Channel.fromPath
bad_snps_ch
report_diffmiss_ch
qc2A_ch
into
cc_ch
compPCA
out_only_pcs_ch
cc2_ch
report_misshet_ch
pcares
drawPCA
report_pca_ch
report_qc1_ch

Process	Tot hours	% times	% cpu number used (Mean)	Max mem (MB)	NF processes
analyseX	0.00	1.13	28.40	67.10	1
batchProc	0.00	0.31	106.50	100.70	1
calculateMaf	0.00	0.23	55.10	138.20	1
calculateSampleHeterozygosity	0.10	2.40	25.70	105.90	1
calculateSnpSkewStatus	0.00	1.03	295.90	212.60	1
compPCA	0.60	19.73	365.40	1200.00	1
drawPCA	0.00	0.54	85.70	83.60	1
findHWEofSNPs	0.00	0.98	5.30	2.40	1
findRelatedIndiv	0.00	0.40	32.80	49.70	1
findSnpExtremeDifferentialMissingness	0.00	0.33	101.30	125.50	1
generateDifferentialMissingnessPlot	0.00	0.33	107.60	327.70	1
generateHwePlot	0.00	0.91	54.30	344.20	1
generateIndivMissingnessPlot	0.00	0.33	136.20	11.40	1
generateMafPlot	0.00	0.42	76.20	512.40	1
generateMissHetPlot	0.00	1.07	90.50	101.90	1
generateSnpMissingnessPlot	0.10	2.54	57.10	299.00	1
getBadIndivsMissingHet	0.00	1.41	61.70	49.70	1
getDuplicateMarkers	0.00	1.64	82.40	221.60	1
getInitMAF	0.10	2.55	10.80	171.80	1
getX	0.10	2.40	17.30	113.90	1
identifyIndivDiscSexinfo	0.10	3.33	25.70	214.90	1
inMD5	0.00	1.64	23.10	8.10	1
noSampleSheet	0.00	1.30	34.10	61.80	1
outMD5	0.00	0.95	35.30	8.10	1
produceReports	0.00	1.37	4.60	25.10	1
pruneForIBDLD	0.90	31.43	384.50	1500.00	1
removeDuplicateSNPs	0.10	4.17	15.30	199.50	1
removeQCIndivs	0.00	1.60	28.20	152.20	1
removeQCPhase1	0.30	10.20	28.00	196.30	1
removeSkewSnps	0.00	0.69	47.80	152.40	1
showHWEStats	0.00	1.01	76.70	926.10	1
showInitMAF	0.00	1.62	51.40	484.80	1

Table 4: Statistics resumé of the QC workflow: process – Nextflow process name; Tot. hours – total hours used by NF process; % times – percentage of times used by process compared to other process ; % cpu number used (Mean) – mean % cpu number used by the process; Max mem (MB) is maximum of memory (resident set size) used by one process; NF processes – number of Nextflow process used for the steps

3.2 Association

- input :
  - phenotype file and one or more phenotype, covariates
  - genetics data in plink file and in option dosage : bgen (regenie, SAIGE, fastGWA and BOLT-LMM), VCF (SAIGE) and impute2 (BOLT-LMM)
- output :
  - each summary statistics of each software and phenotype used and pdf report contains for each combination 3
  - report with Manhattan, qq plot and best result
  - relatedness comptued for each software

3.3 Fine-mapping

- Objective: apply a fine-mapping on significant regions of summary statistics result
- Input data : summary statistics, causal variant number and genetics data in PLINK format
- identify region to apply fine-mapping on full summary statistics using PLINK clump to identify lead SNPs.
- Steps
  - for each region apply different algorithms to find the number of independent SNPs, putative causal variant and credible interval with different software for fine-mapping : COJO (step-wise model selection procedure to select independently associated), FINEMAP (stochastic and conditional algorithm), Caviarbf and PAINTOR software with possibility to use eQTL information.
- Output 4:
  - intermediate file produce in workflow and by each software
  - file contains all result merge
  - figures plot as locus zoom with has been had probability of each software of fine-mapping.
- Test : Fine-mapping has been done on summary statistics obtained with cholesterol output of association testing done with GEMMA with AWI-Gen data set. We obtained 40 windows with $p < 5 \times 10^{-8}$: for each region different software was used fine-mapping

Figure 5 gives an overview of the execution and Table 5 shows the detailed computational costs.

Process	Tot hours	% times	% cpu number used (Mean)	Max mem (MB)	NF processes
clump_data	0.20	2.19	61.60	3900.00	1
ComputedCaviarBF	0.50	5.79	86.80	16.50	40
ComputedCojo	0.50	5.54	76.41	11.10	40
ComputedFineMapCond	0.50	6.72	81.09	11.20	40
ComputedFineMapSSS	0.40	5.53	164.14	8.20	40
ComputedLd	0.90	11.41	113.56	38.40	40
ComputedPaintor	0.40	5.29	85.59	76.40	40
extract_sigpos	0.00	0.12	131.90		1
ExtractPositionGWAS	1.90	22.84	95.63	3800.00	40
GetGenesInfo	0.10	1.23	42.60	2000.00	1
GWASCatDl	0.00	0.25	77.30	445.20	1
MergeResult	0.70	8.12	93.01	439.10	40
SubPLINK	2.00	25.00	78.50	826.50	40

Table 5: Statistics resumé of fine-mapping workflow running on cluster, using cholesterol phenotype. Process - it is Nextflow process name; Tot. hours – total hours used by NF process; % times – percentage of times used by process compared to other process ; % cpu number used (Mean) - mean % cpu number used by the process; Max mem (MB) is maximum of memory used by one process; NF processes - number of Nextflow process used for the steps

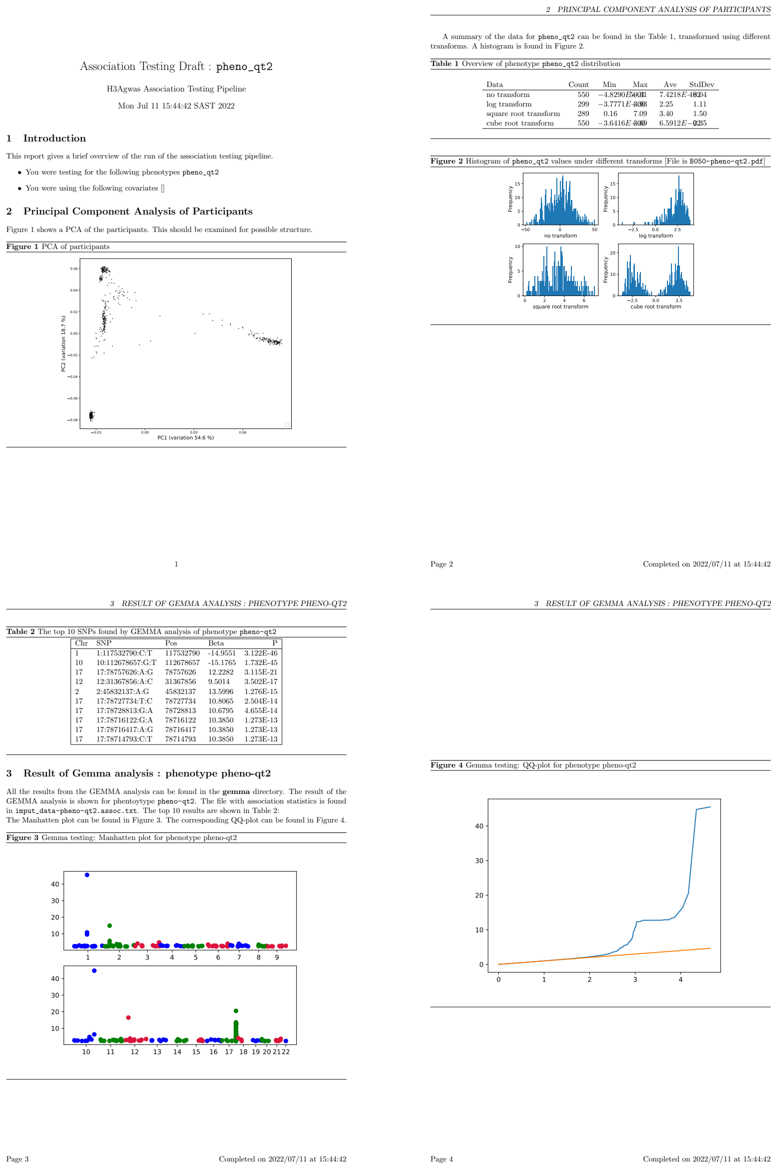

Figure 3: Example of four first pages of association report generated by pipeline with 10 best solutions, qq plot and manhantan plot

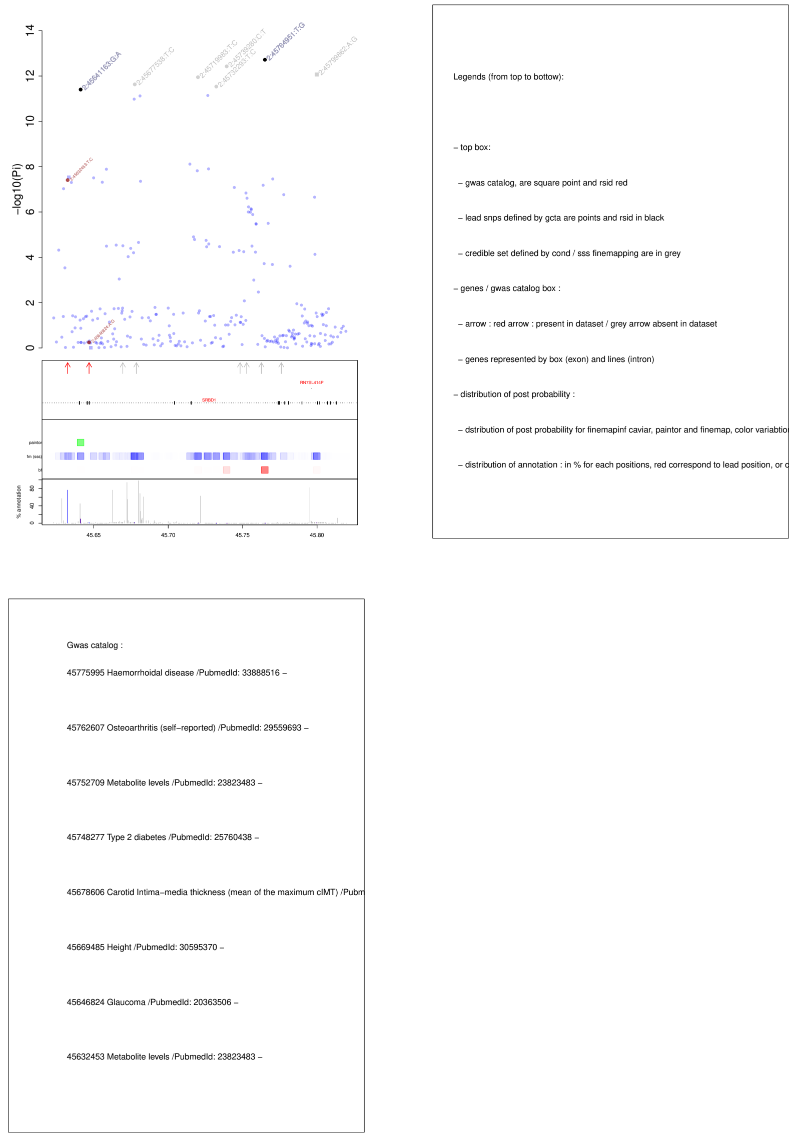

Figure 4: Example of report generated by fine-mapping pipeline contained page 1)locus-zoom with lead snps defined using stepwise model selection procedure (gcta), credible position from fine-mapping softwares, post-probability of fine-mapping software, information relative to GWAS catalog, genes. Pages 2 : legends. Pages 3 : GWAS catalog information's.

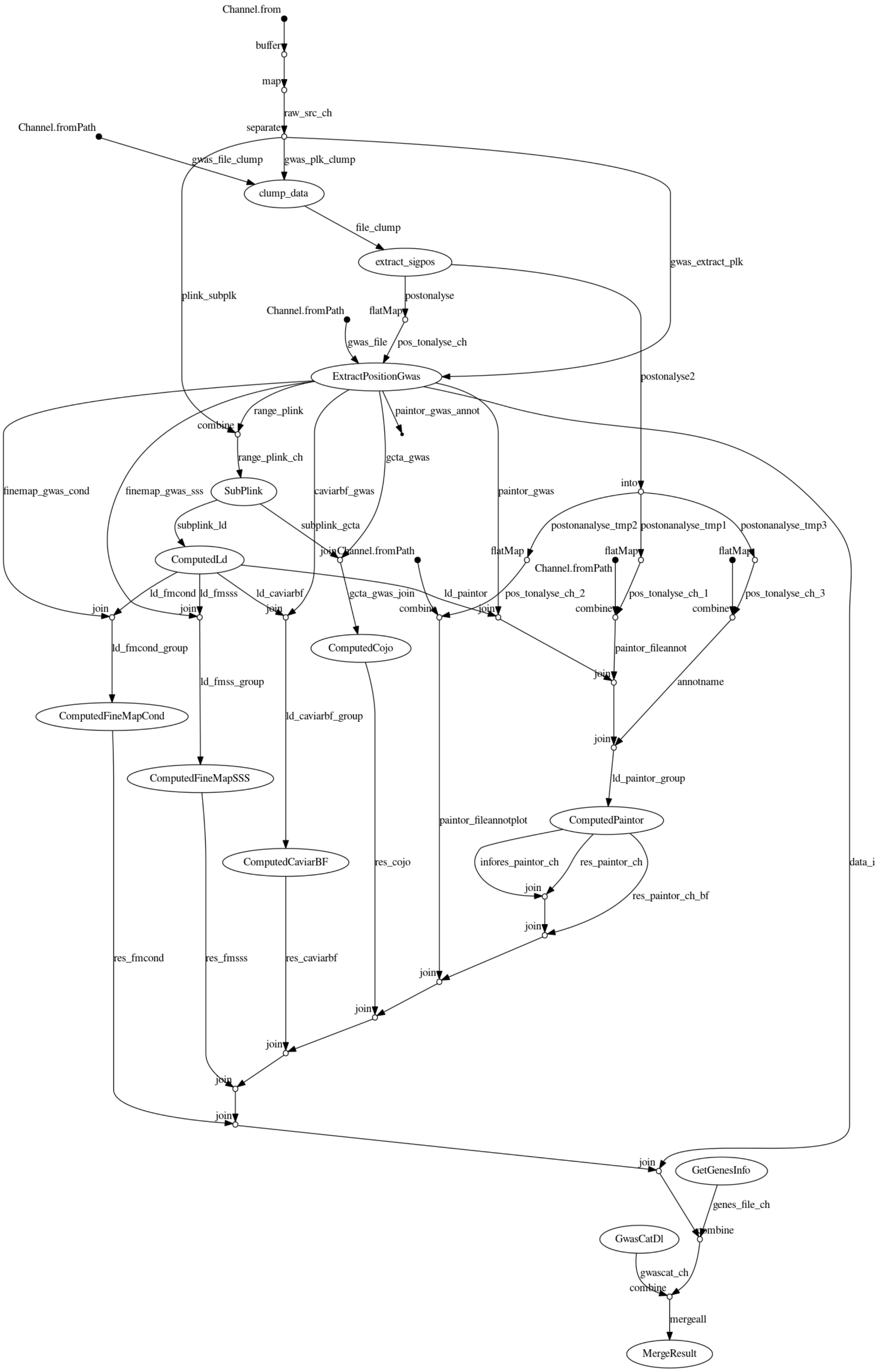

Figure 5: flowchart of Fine-mapping workflow

3.4 Heritability

- Objectives : compute heritability and/or co-heritability using genetics diversity and phenotype or/and summary statistics
- Input : genetics data and phenotype or/and summary statistics.
- Steps :
  - format and prepared files
  - build matrix of relatedness or/and genetic relationships matrix for GCTA, GEMMA
  - computed heritability using GEMMA and LDSC using summary statistics and GCTA, GEMMA and BOLT-LMM using genetics and phenotype.
- Test: using 4 phenotypes of lipid, genotype and summary statistics obtained using association testing result, we ran heritability and co-heritability using the cluster.
- Output : pipeline gave all intermediate file from each software but also a barplot with each heritability (see Figure 6)

An overview is shown in Figure 7 and the detailed computational cost is shown in Table 6.

Process	Tot hours	% times	% cpu number used (Mean)	Max mem (MB)	NF processes
doGemmah2	4.10	4.26	372.19	4200.00	8
doGemmah2_Stat	0.00	0.01	67.39	43.00	8
DoGemmah2Pval	59.50	61.55	894.45	8300.00	4
doGemmah2Pval_Stat	0.00	0.00	63.38	43.00	4
doGRLEM_GCTA_Stat_multi	0.00	0.00	78.15	1.90	4
doh2Bolt	6.10	6.34	1523.67	5700.00	4
doh2Bolt_Stat	0.00	0.01	70.15	43.00	4
doh2BoltiMulti	17.00	17.61	1973.90	5700.00	1
DoLDSC	0.40	0.44	98.10	13900.00	1
doLDSC_Stat	0.00	0.00	29.50	43.00	1
doMultiGRM	0.50	0.55	779.73	5800.00	4
GCTAComputeMultiGRM	0.50	0.51	495.40	6500.00	1
GCTAGRMByFile	7.40	7.66	99.40	2700.00	4
GCTAStrat	0.00	0.00	79.00	158.00	1
getBoltPhenosCovar	0.00	0.01	47.60	65.30	1
getGctaPhenosCovar	0.00	0.04	44.80	66.60	4
getGemmaRel	0.80	0.82	673.60	5100.00	1
MergeFile	0.00	0.00	31.20		1
MergeH2	0.00	0.01	64.40	106.80	1
select_rs_format	0.20	0.17	54.40	609.70	1

Table 6: Statistics resume of heritability workflow running with cluster, using 4 phenotypes and 10,000 individuals and corresponding summary statistics. Process – Nextflow process name; Tot. hours – total hours used by NF process; % times – percentage of times used by process compared to other process ; % cpu number used (Mean) – mean % cpu number used by the process; Max mem (MB) is maximum of memory (RSS) used by any process; NF processes – number of Nextflow processes used for the steps.

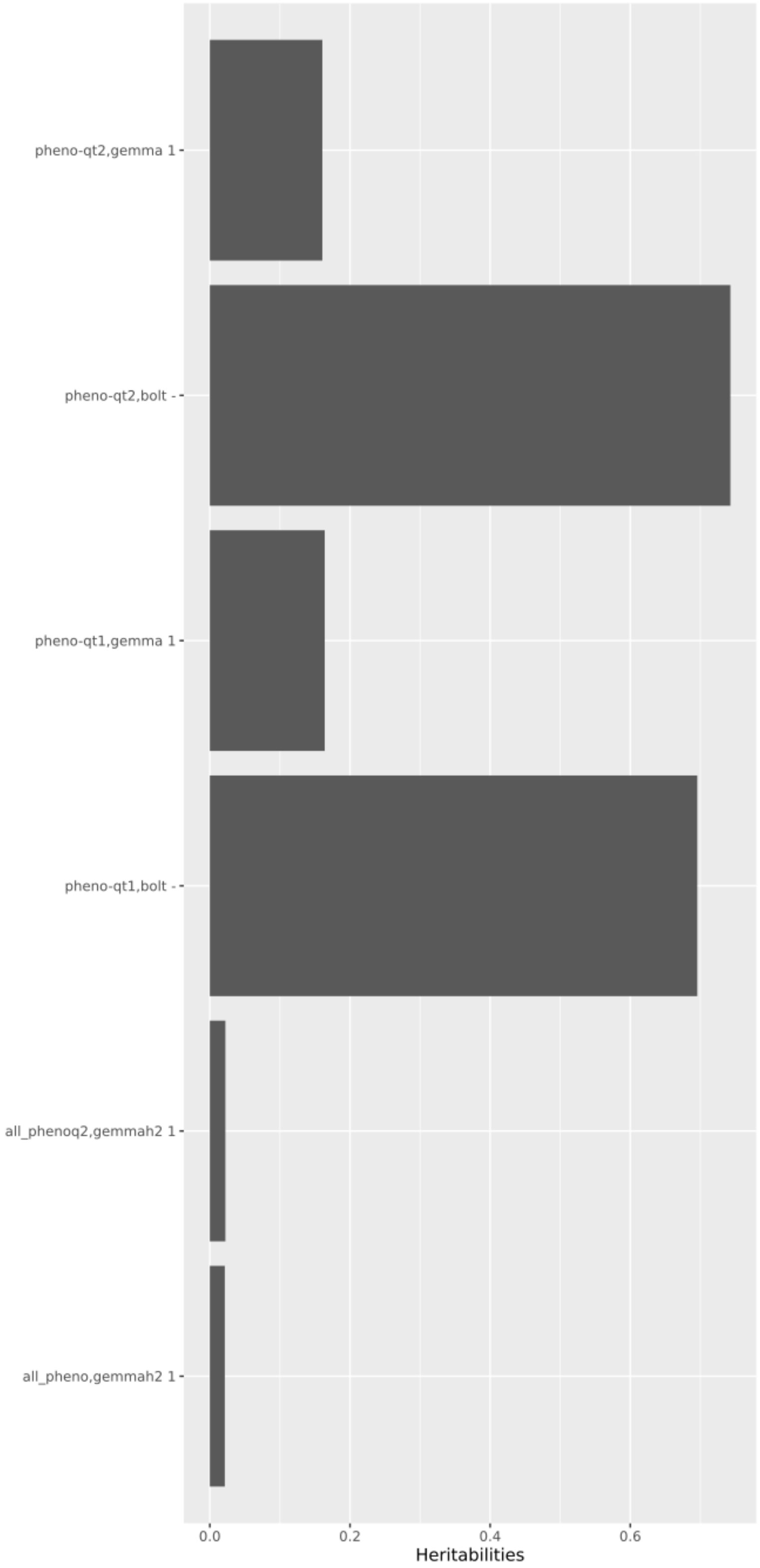

Figure 6: example of output of heritability workflow

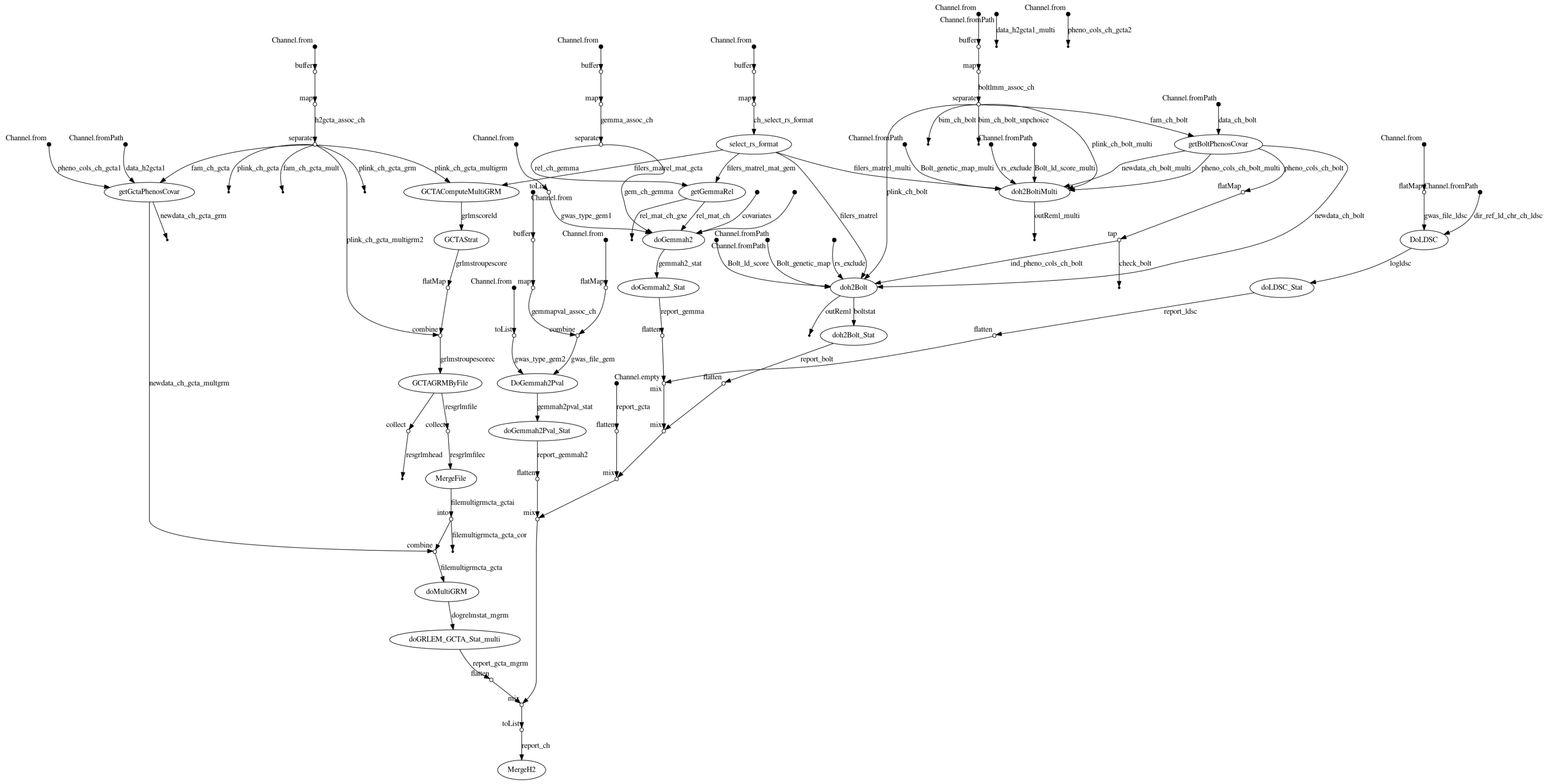

Figure 7: flowchart of heritability workflow

3.5 Simulations workflow

Three workflows of simulation exist

3.5.1 Description of each workflow

Workflow	Input	genotype	Simulation process	Output	Post-simulation computation
`utils/build_example_data/main.nf`	• genotype in VCF file (or downloaded by ftp, by default 1000 Genomes, v37),<br>• effect database (or download by ftp GWAS catalog), phenotype of database,<br>• positions reference in BED format as array positions<br>• phenotype of effect database	Extract positions reference from genetics data, clean and format in plink file	extract positions and effect from the Effect database, extract corresponding genotype and simulated phenotypes with GCTA	• genetic data of population<br>• phenotypes quantitative or qualitative	Randomly switch the sex of some individuals to test `qc` pipeline
`utils/build_example_data/simul-assoc_gcta.nf`	• genotype in plink file<br>• effect database<br>• phenotype of effect database	None	as above	phenotypes quantitative or qualitative	None
`utils/build_example_data/simul-assoc_phenosim.nf`	genotype in plink file	None	random positions will be selected and phenotype simulated using *phenosim*	• phenotype simulated<br>• summary statistics of associations and statistics (FP and TP)	GEMMA and BOLT-LMM will run on genetics data and phenotype simulated. False Positive, True positive rate will be computed

Table 7: Description : input - Input of different pipeline, genotype - if pipeline produce also a independent genotype complementary of phenotype simulation; simulation - how pipeline simulated phenotype; output - what output give pipeline; Post-simulation computation - Analyse or modification done after simulations

3.5.2 Simulation using 1000Genome, GWAS catalog and GCTA

- Objectives : building phenotype using genetics data
- Input : by default, workflow uses (1) genetics data from 1000 Genomes Project (2) result of lead SNPs from GWAS catalog (3) list of phenotype choice in GWAS catalog to build phenotypes.
- Steps
  - Downloads GWAS catalog.
  - Extracts and format GWAS catalog file with extraction of positions and effect using list of SNPs.

– Downloads genomics data of positions extracted from GWAS catalog and array.
– Extracts independent positions from position of GWAS catalog using "–clump" of PLINK and genetic data download.
– Uses Genomics data and z values extracted from GWAS catalog using independent positions to build phenotype using GCTA.
– Output :
  * genotype in plink format of positions from array defined in input.
  * Quantitative and qualitative phenotype with position and genotype used to build phenotype corresponding and information relative to GWAS catalog and used to build phenotype.

- Test : build phenotypes using data of 1000 Genomes project, GWAS catalog and *diabetes* as phenotype.

See Table 8 for the detailed computational costs and Figure 8 for an overview of the different steps.

Process	Tot hours	% times	% cpu number used (Mean)	Max mem (MB)	NF processes
addSexFile	0.00	0.01	22.00	3.90	1
cleanPLINKFile	0.20	0.26	417.94	1.50	22
cleanPLINKFile_GC	0.40	0.56	307.69	1.50	22
Dl1000G	52.10	75.30	1.50	38.30	22
Dl1000G_GC	3.90	5.71	0.95	13.80	22
format_sim_qualitatif	0.00	0.03	90.30	20.90	1
format_sim_quantitatif	0.00	0.03	88.80	15.10	1
format_simulated	0.00	0.01	91.30	13.70	1
getchr_gc	0.00	0.03	39.30		1
GWASCatDl	0.10	0.11	75.30	245.30	1
mergePLINKFile	0.00	0.01	135.00	71.20	1
mergePLINKFile_GC	0.00	0.01	307.40	12.70	1
simulation_qualitatif	0.00	0.01	89.10	1.50	1
simulation_quantitatif	0.00	0.01	95.00	5.60	1
transfvcfInBed1000G	2.10	3.11	422.98	9.20	22
transfvcfInBed1000G_GC	10.20	14.79	530.84	1.50	22

Table 8: Summary result for simulation individual using GWAS catalog for phenotype and 1000 genome project as genotype data. Process - it is Nextflow process name; Tot. hours – total hours used by NF process; % times – percentage of times used by process compared to other process ; % cpu number used (Mean) - mean % cpu number used by the process; Max mem (MB) is maximum of memory used by one process; NF processes - number of Nextflow process used for the steps

3.6 Format data

3.6.1 Convert PLINK format to VCF

- Objective : converts data to VCF for imputation
- Input PLINK file, reference genome of FASTA file and reference for positions, chromosome, and rs name.

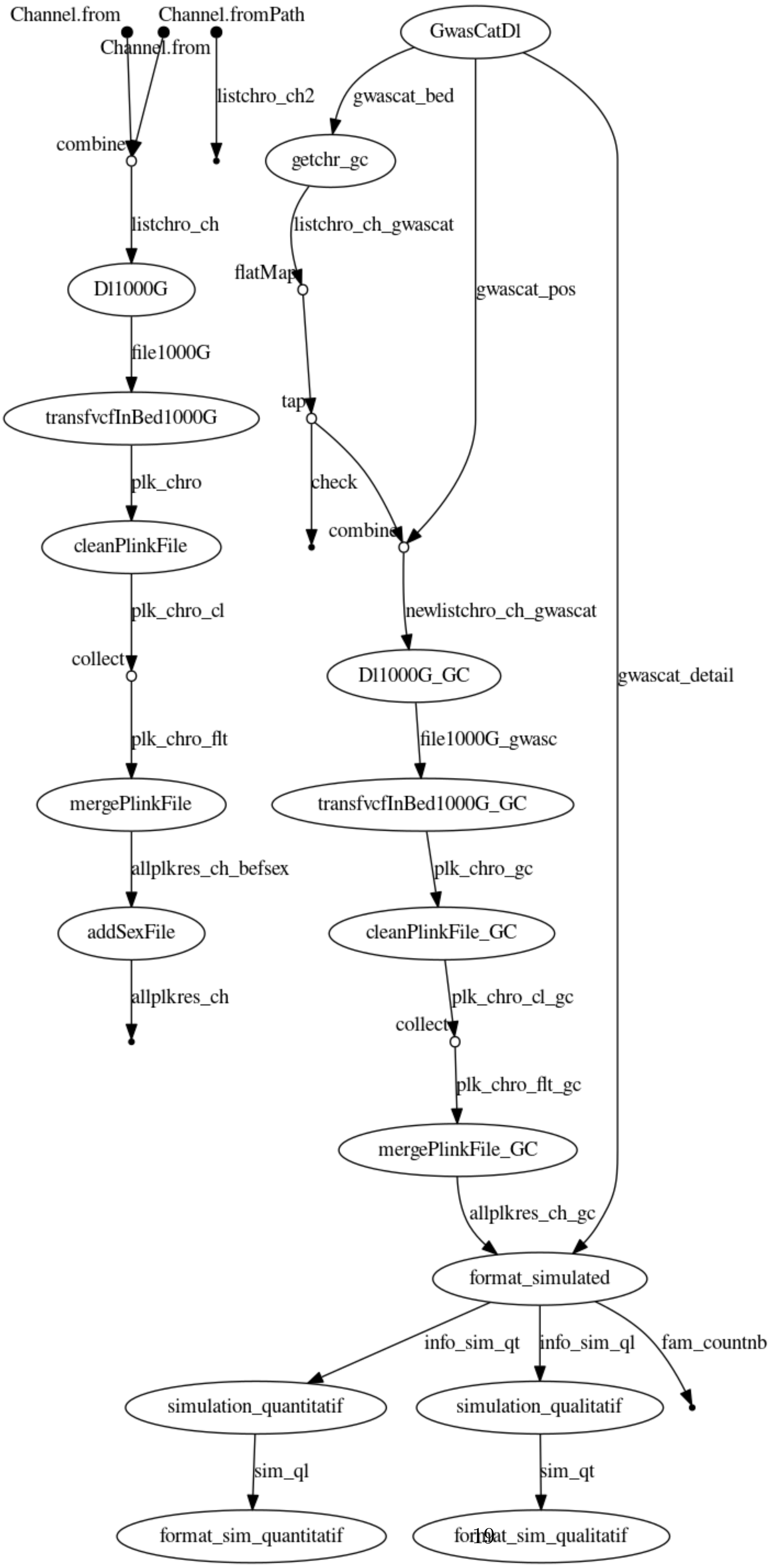

Figure 8: flowchart of simulation workflow

- Steps
  - extract rsid and information of each positions from the reference file and reference sequence.
  - splits file by chromosome (optional)
  - converts PLINK to VCF.
  - cleans and rename position name.
  - fix allele using BCFtools
- Output : VCF file and VCF file by chromosome.
- Test : convert data after QC of 10,796 individuals using workflow in VCF format

See Figure 9 for an overview of the process and Table 9 for the detailed computational costs.

Process	Tot hours	% times	% cpu number used (Mean)	Max mem (MB)	NF processes
checkfixref	0.40	7.73	98.80	11.00	1
checkVCF	0.40	8.12	97.40	173.40	1
convertInVcfChro	3.00	62.03	155.99	1100.00	22
convertrsname	0.00	0.43	86.50	211.60	1
CounChro	0.10	1.29	3.50	2.60	1
deletedmultianddel	0.00	0.63	93.30	687.60	1
extractpositionfasta	0.00	0.19	50.90	1.50	1
extractrsname	0.70	13.46	98.60	505.60	1
mergevcf	0.20	3.86	319.00	16.00	1
refallele	0.10	2.26	13.80	165.60	1

Table 9: Summary of the result of the workflow to format PLINK to VCF prepare data for imputation. Process – Nextflow process name; Tot. hours – total hours used by NF process; % times – percentage of times used by process compared to other process; % cpu number used (Mean) – mean % cpu number used by the process; Max mem (MB) is maximum of memory used by one process (resident set size); NF processes – number of Nextflow processes used for the steps

3.6.2 Convert VCF to PLINK or other format

- Objective : convert output from imputation in PLINK or other format to run association testing.
- Input : list of VCF, genetic map.
- Steps
  - Computed various statistics as frequency and imputation score
  - Filter VCF by score and frequency
  - Transform file to PLINK.
  - check for rs duplicate and correct.
  - merge all files PLINK by chromosome.

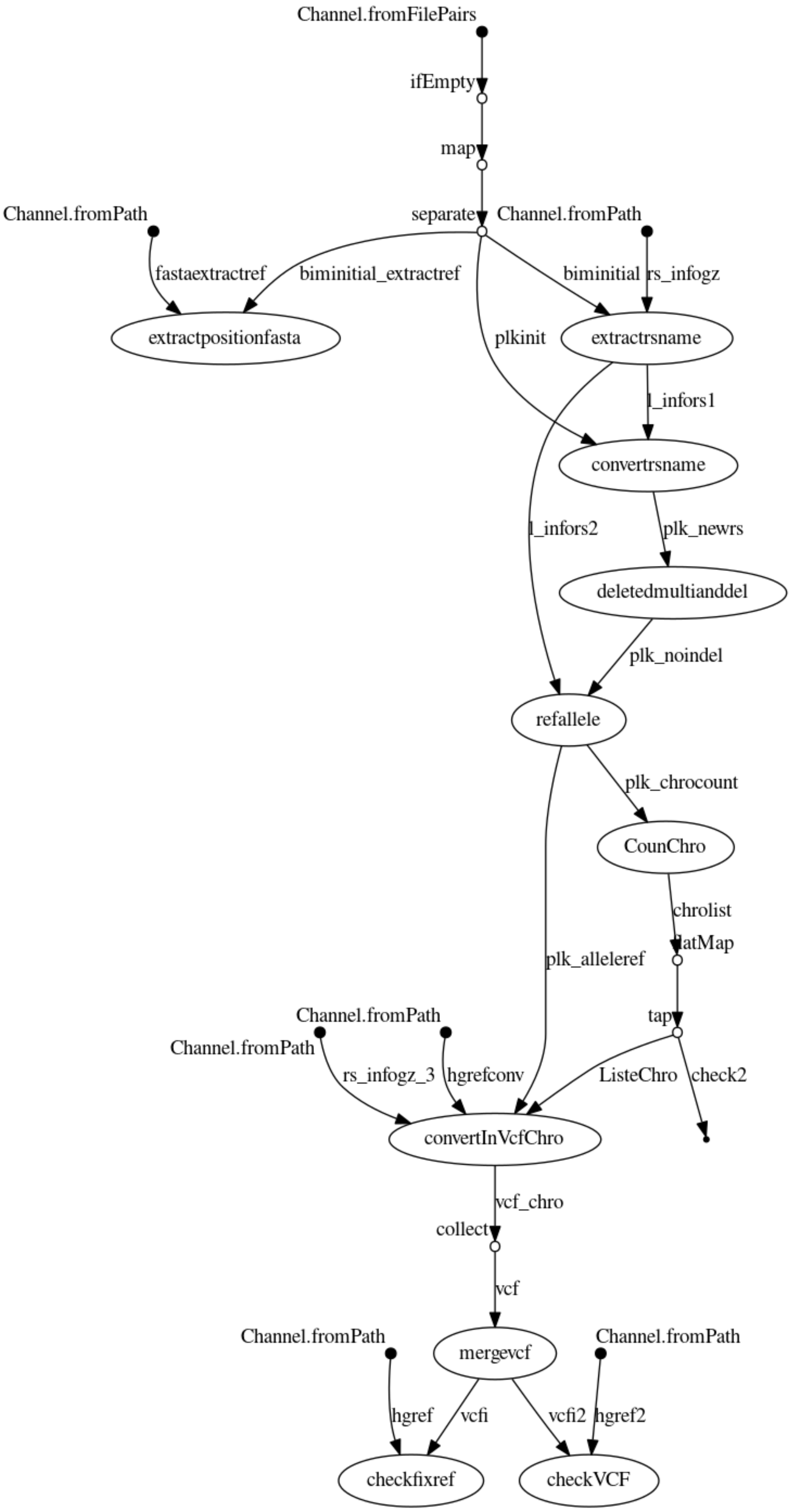

Figure 9: Flowchart of workflow to convert PLINK format to VCF to prepare data for imputation

- Output: PLINK file and report presenting distribution of quality and frequencies 10 and plink files converted.
- Test : used output of imputed data (≈30 millions SNPs and 12,000 individuals)

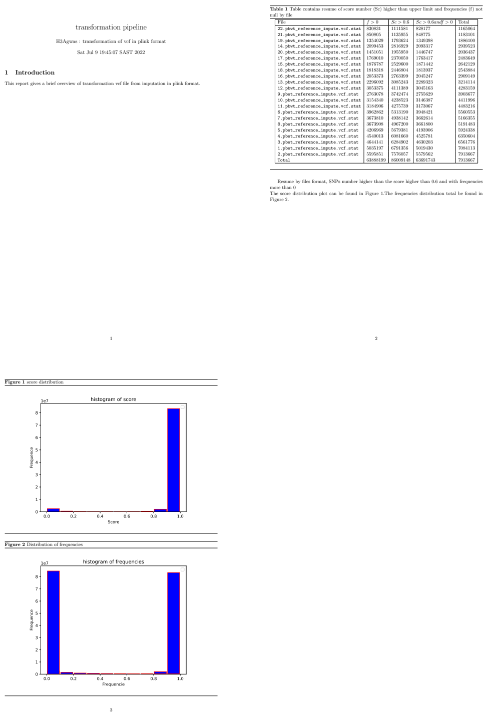

transformation pipeline

H3Agwas : transformation of vcf in plink format

Sat Jul 9 19:45:07 SAST 2022

1 Introduction

This report gives a brief overview of transformation vcf file from imputation in plink format.

1

Table 1 Table contains resume of score number (Sc) higher than upper limit and frequencies (f) not null by file

File	$f > 0$	$Sc > 0.6$	$Sc > 0.6 and f > 0$	Total
22.pbwt_reference_impute.vcf.stat	830831	1111581	828177	1165064
21.pbwt_reference_impute.vcf.stat	850805	1135955	848775	1183101
19.pbwt_reference_impute.vcf.stat	1354029	1793624	1349398	1886100
14.pbwt_reference_impute.vcf.stat	2099453	2816929	2093317	2939523
20.pbwt_reference_impute.vcf.stat	1451051	1955950	1446747	2036437
17.pbwt_reference_impute.vcf.stat	1769010	2370050	1763417	2483649
15.pbwt_reference_impute.vcf.stat	1876787	2529600	1871442	2642129
18.pbwt_reference_impute.vcf.stat	1818318	2446804	1813937	2543884
16.pbwt_reference_impute.vcf.stat	2053373	2763399	2045247	2909149
13.pbwt_reference_impute.vcf.stat	2296092	3085243	2289323	3214114
12.pbwt_reference_impute.vcf.stat	3053375	4111389	3045163	4283159
9.pbwt_reference_impute.vcf.stat	2763078	3742474	2755629	3903677
10.pbwt_reference_impute.vcf.stat	3154340	4238523	3146387	4411996
11.pbwt_reference_impute.vcf.stat	3184906	4275739	3173067	4483216
6.pbwt_reference_impute.vcf.stat	3962862	5313190	3948421	5560553
7.pbwt_reference_impute.vcf.stat	3673810	4938142	3662614	5166355
8.pbwt_reference_impute.vcf.stat	3673908	4967200	3661800	5191483
5.pbwt_reference_impute.vcf.stat	4206969	5679381	4193906	5924338
4.pbwt_reference_impute.vcf.stat	4540013	6081660	4525781	6350604
3.pbwt_reference_impute.vcf.stat	4644141	6284902	4630203	6561776
1.pbwt_reference_impute.vcf.stat	5035197	6791356	5019430	7084113
2.pbwt_reference_impute.vcf.stat	5595851	7576057	5579562	7913667
Total	63888199	86009148	63691743	7913667

Resume by files format, SNPs number higher than the score higher than 0.6 and with frequencies more than 0

The score distribution plot can be found in Figure 1.The frequencies distribution total be found in Figure 2.

2

Figure 1 score distribution

Figure 2 Distribution of frequencies

3

Figure 10: Output of pipeline format vcf in plink, with distribution of quality and frequency (figures with general distribution and table distribution by frequency)

An overview is shown in Figure 11 and the detailed computational costs are shown in Table 10.

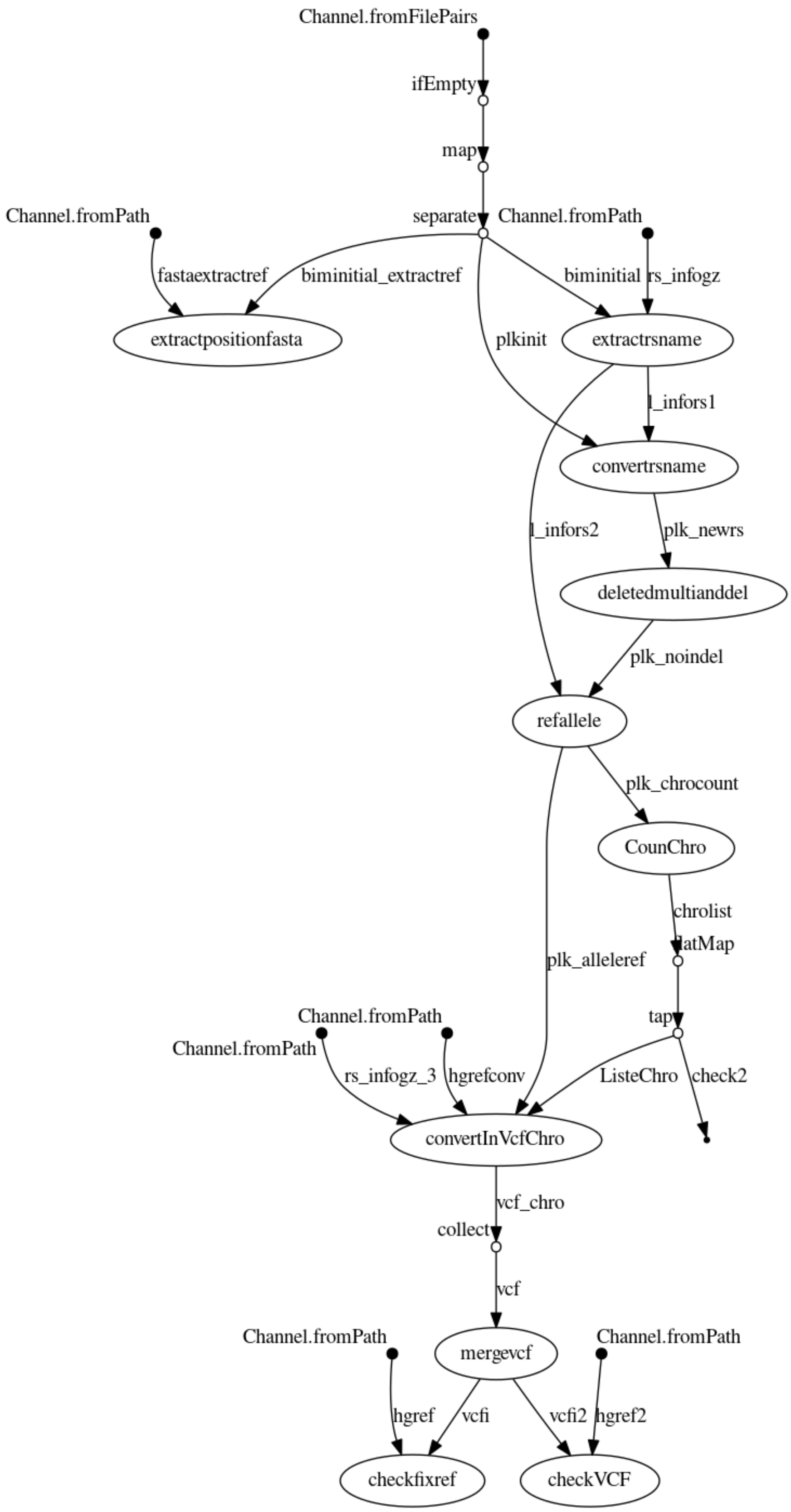

Figure 11: flowchart of workflow to convert VCF file from imputation into PLINK format

Process	Tot hours	% times	% cpu number used (Mean)	Max mem (MB)	NF processes
TransformRsDup	7.20	4.71	10.97	224.70	22
AddedCM	2.80	1.83	26.99	187.50	22
computedstat	13.20	8.65	99.33	8.60	22
dostat	0.10	0.08	96.80	6400.00	1
formatvcfscore	128.00	84.08	108.37	196.10	22
GetRsDup	0.00	0.02	89.00	2900.00	1
MergePLINK	1.00	0.64	83.10	62700.00	1

Table 10: Summary of result of workflow to format VCF after imputation in PLINK format using data after imputation obtained in QC. Process – Nextflow process name; Tot. hours – total hours used by NF process; % times – percentage of times used by process compared to other process ; % cpu number used (Mean) – mean % cpu number used by the process; Max mem (MB) is maximum of memory used by one process; NF processes – number of Nextflow processes used for the steps.

3.6.3 Multi-trait analyse using MTAG

- Objective : Analyse multi trait using summary statistics
- Input : result of summary statistics from various phenotype
- Steps
  - format each files to prepared input for MTAG software
  - Run MTAG with all summary statistics
  - Run MTAG seletected 2 by 2 each summary statistics.
- Output : result of mtag sofware (summary statistics), and report in PDF as association pipeline
- Test : used output summary statistics (14 millions SNPs) for 4 SNPs

An overview is shown in Figure 12 and the detailed computational costs in Table 11.

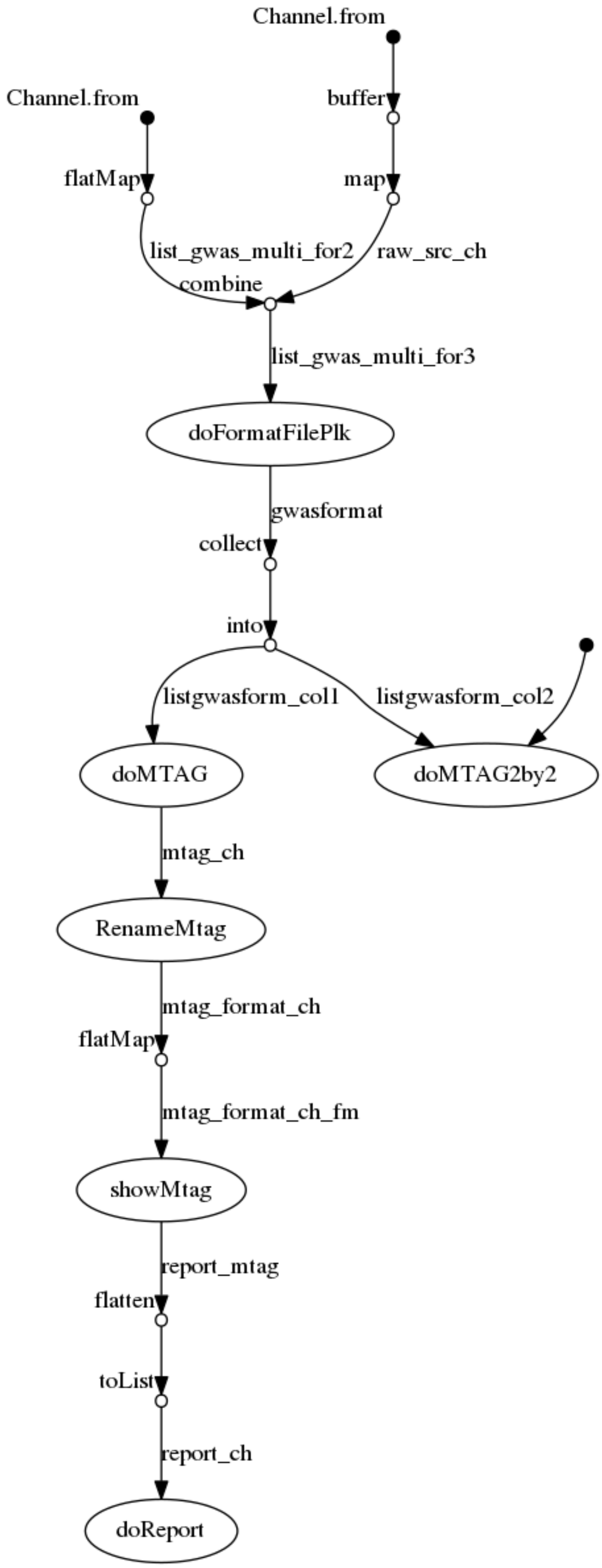

Figure 12: flowchart of workflow to convert VCF file from imputation in PLINK format

Process	Tot hours	% times	% cpu number used (Mean)	Max mem (MB)	NF processes
doFormatFilePlk	0.70	13.13	71.35	6400.00	4
doMTAG	1.20	22.82	98.70	34600.00	1
doMTAG2by2	3.00	59.39	100.35	18600.00	6
doReport	0.00	0.02	45.10	20.50	1
RenameMtag	0.00	0.88	17.70	9.30	1
showMtag	0.20	3.76	91.75	6900.00	4

Table 11: Summary of result of workflow doing a multi trait analysis using MTAG: Process – Nextflow process name; Tot. hours – total hours used by NF process; % times – percentage of times used by process compared to other process; % cpu number used (Mean) – mean % cpu number used by the process; Max mem (MB) is maximum of memory used by one process; NF processes – number of Nextflow processes used for the steps.

4 Detail of experimentation in the main paper

These tables give detail of the experimentation described in the main paper.

Process	Tot hours	% times	% cpu number used (Mean)	Max mem (MB)	NF processes
computePCA	2.3	0.5	374	1100	1
drawPCA	0.0	0.0	114	89	1
extractPheno	0.0	0.0	81	63	1
bgen_formatsample	0.0	0.0	92	93	1
indexbgen_list	3.4	0.8	41	7	22
getBoltPhenosCovar	0.0	0.0	79	68	1
select_rs_format	0.0	0.0	58	635	1
FastGWADoGRM	11.5	2.7	227	2100	100
MergFastGWADoGRM	0.0	0.0	17	3	1
computeTest	8.1	1.9	100	824	4
format_genetic_ldscore	0.4	0.1	43	2200	1
doBoltmm	5.7	1.3	351	3600	4
getListeChroGem	0.0	0.0	120	5	1
getGemmaRel	0.6	0.1	854	5100	1
doGemmaChro	312.1	72.7	945	5400	88
doMergeGemma	0.1	0.0	24	4	4
FastGWARun	41.9	9.8	972	2200	4
getListeChro_saige	0.0	0.0	81	5	1
getchrobgen	0.0	0.0	86	23	22
getSaigePheno	0.0	0.0	103	66	1
checkidd_saige	0.0	0.0	43	136	1
subplink_heritability_saige	0.0	0.0	74	927	1
saige_computed_variance	1.5	0.4	878	806	4
doSaigeListBgen	13.4	3.1	99	644	88
doMergeSaige	0.0	0.0	40	4	4
regenie_step1	4.6	1.1	176	5900	4
regenie_step2	22.6	5.3	925	373	88
merge_regenie	0.1	0.0	24	4	4
format_regeniesumstat	0.1	0.0	78	10	4
ShowManhattan	0.7	0.2	97	7900	20
drawPlinkResults	0.1	0.0	83	4300	4
showPhenoDistrib	0.0	0.0	85	127	1
doReport	0.0	0.0	34	25	1

Table 12: Cost of associationon cluster, using 4 phenotypes and 10,700 individuals. The elapsed time for the entire workflow was 12h 36min, with a high degree of parallelisation. *Process* is the Nextflow process name; *Tot hours* – total CPU hours used by instances of this NF process; *% times* – % of time used by process compared to other process; *% cpu number used (Mean)* – mean % cpu number used by instances of the process — a measure of achievable parallelism for instances of that process; *Max mem (MB)* is the maximum resident set size used by one process; *NF processes* – number of Nextflow process used for the steps, and a measure of parallelism at very coarse level.

Process	Tot hours	% times	% cpu number used (Mean)	Max mem (MB)	NF processes
GetRsFile	1.1	1.2	99.5	9	1
ChangeFormatFile	29.9	30.7	65.2	4100	3
doGWAMA	18.0	18.5	67.4	10400	1
doMetal	2.9	3.0	99.6	3500	1
doMetaSoft	12.3	12.7	103.1	7700	1
doMRMEGA	15.8	16.2	68.1	8100	1
doPlinkMeta	8.9	9.2	99.8	2000	1
showGWAMA	1.4	1.5	100.2	5800	1
showMetal	1.8	1.8	97.7	3800	1
showMetasoft	1.9	1.9	100.1	7500	1
showMRMEGA	1.6	1.6	100.2	6200	1
showPlink	1.2	1.3	100.0	4800	1
doReport	0.6	0.6	46.1	24	1

Table 13: Cost of running meta-analysis workflow using Wits cluster, using 3 summary statistics and 14 millions of positions by summary statistics. Column labels as in Table 12
